# *In vitro* study of accuracy of subaxial cervical pedicle screw insertion using calipers based on the gravity line

**DOI:** 10.1371/journal.pone.0181324

**Published:** 2017-07-20

**Authors:** Xiang Yao, Shiqing Liu

**Affiliations:** Department of Orthopedics, Renmin Hospital of Wuhan University, Wuhan, Hubei Province, PR China; Harvard Medical School/BIDMC, UNITED STATES

## Abstract

**Object:**

There is a high probability of iatrogenic perforation of the vertebral pedicle wall during the application of subaxial cervical pedicle screw (CPS). The goal of this study was to evaluate the accuracy of CPS insertion at C3-C7 in vitro using novel calipers based on the gravity line.

**Methods:**

Nine cadaveric cervical spines underwent computed tomography scanning and preoperative design. A lateral fluoroscopic view was taken to measure the intra-operative sagittal angle by C-arm with hanging cross structured K-wires. By referring to the gravity line, caliper A was used to locate the entry point, while caliper B was employed to guide the screw insertion. Postoperative CT scans were performed to assess the accuracy of the screw placements, according to the Neo classification.

**Results:**

Overall, 78 (88.6%) of the 88 pedicle screw placements were classified as grade 0 (correct position), 4 (4.5%) were grade 1 (non-critical perforation), 4 (4.5%) were grade 2 (critical perforation), and 2 (2.3%) were grade 3 (critical perforation).

**Conclusions:**

Using our novel calipers and referring to the gravity line was helpful for locating and guiding individual cervical pedicle screw insertions.

## Introduction

Since Abumi et al. first reported the results of cervical pedicle screw (CPS) fixation for traumatic lesion of the subaxial cervical spine, several studies have been performed indicating its superior biomechanical stability over lateral mass screws[1–3]. The ideal placement of the CPS requires an accurate entry point, with an appropriate trajectory angle and screw size, incorrect CPS placement can sometimes result in lethal perforation in the C3–7 region[4–6].

The use of morphological markers to identify the entry point into C3–7 vertebra is insufficient for safe CPS placement because of the variation in the lateral mass or pedicle [7–9]. Instead, an objective instrument to determine the entry point, instead of subjective judgment that depends on experience, is required.

While the transverse angle of the pedicle is easy to obtain in preoperative computed tomography (CT), the real-time, intra-operative sagittal angle is harder to acquire. This is because the superior or inferior vertebral endplate, which is chosen as the reference line in preoperative design[7, 10–12], is useless during the operation. Several assistance techniques (e.g., aiming frame, locator, intra-osseous ultrasound, fluoroscopy navigation, O-arm navigation, Iso-C 3D navigation, CT-based navigation, the patient-specific template system and the robotic system) were developed to obtain higher accuracy and avoid the lethal perforation[3, 13–17]. Regrettably, the extensive application of these navigation systems or instruments is unrealistic as they are often time-consuming, highly costly, and technologically demanding[3, 18, 19]. Thus, despite its downsides of radiation exposure, the assistance of conventional C-arm fluoroscopy is an effective choice for most cervical spine surgeons[20, 21]. It’s meaningful to design an assistant tool to achieve the ideal screw insertion at ideal angles by means of C-arm.

Two novel stainless steel calipers (caliper A and B), that referred to the gravity line instead of the superior or inferior vertebral endplate, were invented by the authors to locate the entry point and guide the stereoscopic insertion of CPS. The purpose of this in vitro study in cadaveric cervical spines was to evaluate the accuracy of subaxial CPS insertion using specially-made calipers and C-arm fluoroscopy.

## Materials and methods

### Cadavers

Nine formalin-fixed Chinese cadaveric cervical spines donated from volunteers were provided by the Department of Human Anatomy of Nanjing Medical University. The institutional review boards of the participating hospital approved the study. The deceased or their next of kin provided written informed consent for the use of the remains in research. None of the transplant donors were from a vulnerable population and all donors or next of kin provided written informed consent that was freely given.

**Fig 1:**
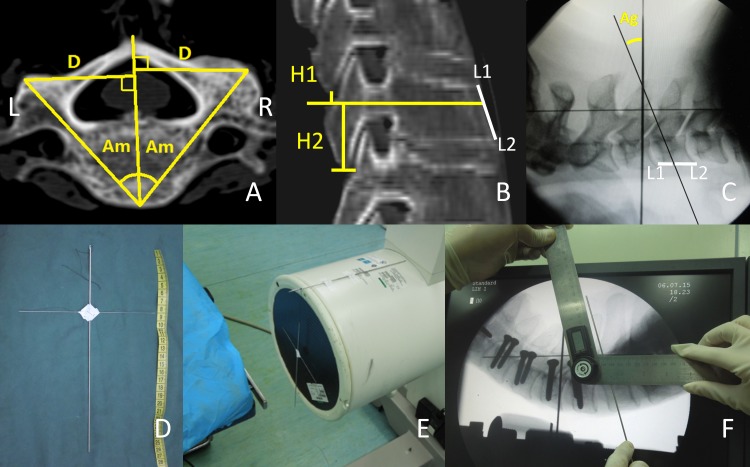
Illustrated methods used to measure all parameters in the study. **(**A) Axial reconstruction image through the pedicle isthmus; D and Am was measured. (B) Oblique sagittal reconstruction image through the longitudinal pedicle axis; H1, H2, L1 and L2 were measured. (C) A lateral fluoroscopy view; Ag was designed with a physical K-wire. (D, E) Cross structured K-wires were free hanging front of the image Intensifier. (F). Ag was read out by an electronic digital goniometer on the monitor.

The spine specimens underwent pre-operative CT scanning using a Siemens 256-slice spiral CT scanner (Philips Medical System, Cleveland, USA), and images (using 1.0mm thick slices) were subsequently transferred and stored digitally on the image workstation (Extended Brilliance Workspace, Philips). A multi-design reconstruction (axial plane, coronal plane and oblique sagittal plane, along the pedicle axis) was carried out, and the design and measurement of the ideal entry point and orientation (Am, D, H1, H2, L1 and L2) in each segment was later determined (Fig 1A and 1B).

Relevant parameters (Fig 1A–1C):

1. Distance (D) [mm]: Vertical distance between entry point and the midline.
2. Pedicle transverse angle (Am): Angle between the longitudinal pedicle axis (LPA) and median plane of vertebral body.
3. Intra-operative pedicle sagittal angle (Ag): Angle between the gravity line and pedicle axis in the sagittal plane. (Ag is negative when the pedicle axis is in the cranial orientation).
4. Height 1 (H1) [mm]: Vertical distance between the LPA and inferior margin of upper lateral mass. (If the entry point was obscured by upper lateral mass, the value is negative).
5. Height 2 (H2) [mm]: Vertical distance between the LPA and inferior margin of lateral mass itself.
6. Length1 (L1) [mm]: Vertical distance between the anterior superior border and the intersection of pedicle axis and vertebral anterior edge.
7. Length2 (L2) [mm]: Vertical distance between the anterior inferior border and the intersection of pedicle axis and vertebral anterior edge.

All assessments were carried out with digital measurement tools in the Workspace of Philips; the averages of the measured values were adopted.

### Calipers

Caliper A and B were designed and made up by the authors using AutoCAD 2011 (Autodesk Computer Aided Design, USA). The calipers obtained a China national Utility Model patent in 2015 (application no.: ZL 2015 2 0449907.6, state intellectual property office of the People’s Republic of China).The first author (X.Y.) is the only patent owner who has potential interest of the device used in the study.

**Fig 2:**
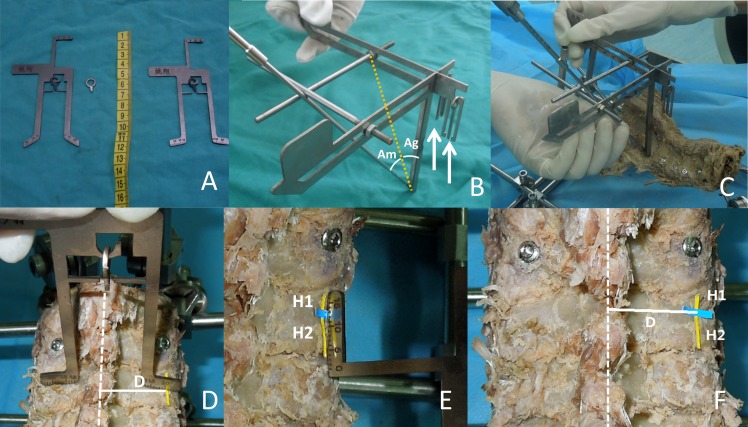
**The physical product of caliper A and B. (**A) caliper A. (B) caliper B, two sliding bars were moved to degrees (Am, Ag) and white arrow showed the rocking indicators. (C) Specimen was fixed and pedicle screw channel was prepared by a manual drill along the connecting line between the intersection of bars and entry point. (D–F) Location of entry point by sequential measurement of D, H1 and H2 using caliper A.

Caliper A has metallic planar structure with a gap where the indicator hangs (Fig 2A). When the gap, indicator, and the spinous process are in a straight line, a furcated structure with a scale was used to measure the vertical distance between the entry point and the midline (D) (range 12–25mm) (Fig 2D). The straight measuring tape on the other end is used to gauge the vertical distance between the LPA and the margin of lateral mass (H1 and H2) (Fig 2E). The entry point was located by sequential measurement of D, H1, and H2 (Fig 2F).

**Fig 3:**
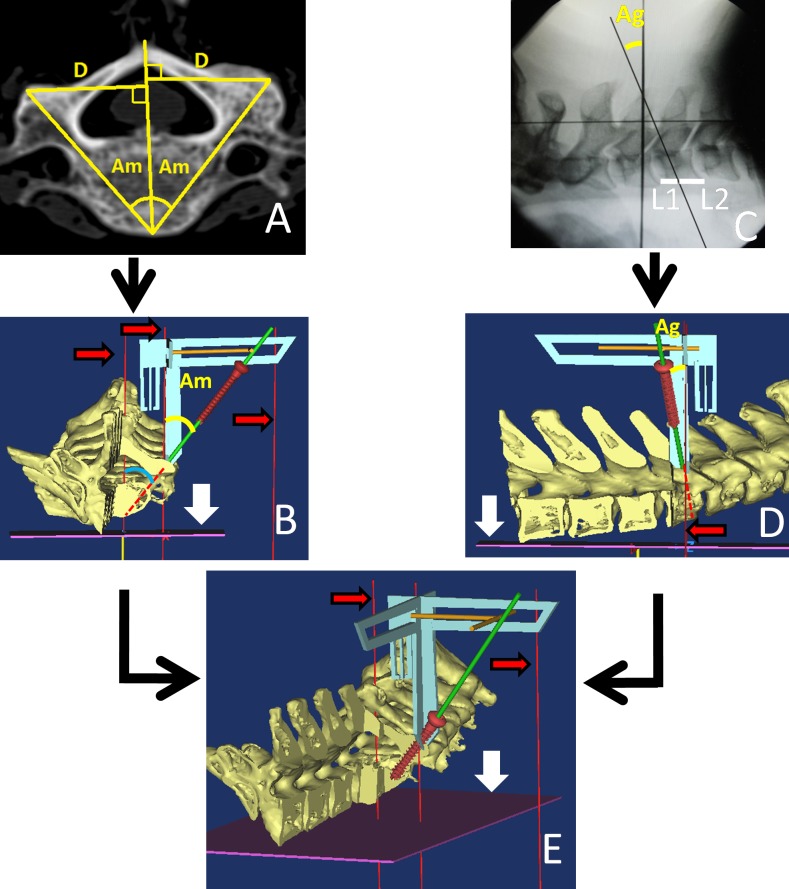
**The three-dimensional demonstration of insertion using caliper A and B.** (A) Am obtained from the preoperative CT. (B) The realization of Am in the coronal plane. (C) Ag obtained from the intraoperative fluoroscopy. (D) The realization of Ag in the sagittal plane. (E) The ideal insertion of pedicle screw in the appropriate orientation. Red narrow arrow: the gravity line. White broad arrow: the ground plane.

Caliper B is a three-dimensional structure welded by two pieces of metallic hollow protractors (range 5–50°), which is horizontally positioned on the entry point (Fig 2B). Two sliding bars were moved to Am (Fig 3A and 3B) and Ag (Fig 3C and 3D), which were measured in advance and met in a point. The connecting line between the intersection of the two bars and the entry point was considered the optimal drill/screw orientation, when the three following conditions were met: one arm parallel to midline, another arm parallel to the coronal plane, and the two rocking indicators mediated between bilateral bulges (Fig 2B, white arrow). As one arm should be placed parallel to the midline by the naked eye, the assistant must hold the caliper as precisely as possible (Fig 2C).

### Screw insertion method

Soft tissue, muscles, and capsules of specimens were completely removed to clearly expose the spinous process and the surface of the lateral mass of C2–C7 vertebra. With the help of caliper A, specimens were fixed horizontally by an external fixator (Fig 2C). Subsequently, standard lateral fluoroscopy was carried out to gain a view containing the cross structured K-wires (Fig 1C–1E). The vertical thick K-wire of the cross structure in the view represented the gravity line (Fig 1C). A physical K-wire was put on the monitor screen at the angle designed preoperatively (referring to the center of the pedicle gallery and the proportion of L1 and L2). Then, the Ag was read out immediately from the electronic digital goniometer (minimum 0.1°) (Fig 1F).

The entry point was located by the sequential measurement of D, H1, and H2 (Fig 2D–2F), where the cortical bone was stabbed by a manual awl. With the two sliding bars marking Am (Fig 3A and 3B) and Ag (Fig 3C and 3D), caliper B was placed on the entry point, and adjusted to meet the three demands mentioned above. The screw channel was prepared by a 2.4 mm manual drill along the connecting line between the intersection of the two bars and the entry point (Fig 2B and 2C). Four walls of the screw channel were inspected for perforation with a flexible bulb-ended probe at various depths and directions. With the 2.0 mm K-wires or probe staying in the hole, standard lateral fluoroscopic view was taken again to avoid manifestation of error. If perforation was detected within the pedicle or in the intra-operative view, the procedure was retried along an adjusted orientation. Partial channels of the specimen with excellent bone quality were tapped, and 3.5 mm screws of appropriate length were subsequently inserted into the made-up bone channels (Fig 3F). Anterior–posterior and lateral view of fluoroscopy of specimens were examined. Considering economic factors, stainless steel screws (with the same diameter and pitch) were used as a substitute for CPS in current study.

### Evaluation of screw position

**Fig 4:**
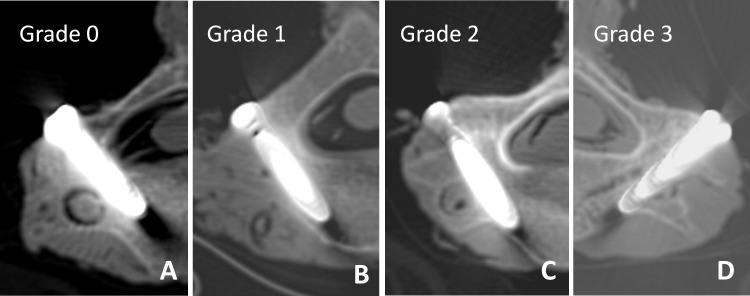
Neo classification of pedicle perforation. **(**A) Grade 0: no perforation. (B) Grade 1: perforation < 2 mm. (C) Grade 2: perforation ≥ 2 and < 4mm. (D) Grade 3: perforation ≥ 4 mm.

Postoperative reconstructed CT scans were performed to assess the screw accuracy according to the previously established Neo classification (Fig 4A–4D)[22] (grade 0: no perforation, grade 1: perforation < 2 mm, grade 2: perforation ≥ 2 and < 4mm, and grade 3: perforation ≥ 4 mm). Grade1 was noncritical perforation, and grade 2 or 3 were considered as critical perforation. The direction of cortical perforation was classified as medial, lateral, superior, or inferior.

### Statistical analysis

Statistical analysis was carried out using the software of Statistical Program for Social Sciences 16.0 (SPSS 16.0, USA). Linear and angular pedicle measurements (D, H1, H2, and Am) were calculated as mean ± stand deviation and compared bilaterally using the paired t-test in each vertebral level. Enumerated data about perforation were displayed as percentage. *P* < 0.05 was considered statistically significant.

## Results

8 specimens contained intact C3–C7 vertebrae and the last specimen contained C4–C7 vertebrae only, 88 pedicles of 44 cervical vertebrae were included in the study. The important linear and angular parameters for the preoperative design (D, Am, Ag, H1, and H2) of all 88 pedicles are displayed in Table 1.

**Table 1:** Relevant parameters of C3-7 pedicles for preoperative design (mean ± stand deviation).

		C3	C4	C5	C6	C7
D(mm)	left(n = 8)	20.4±0.8	20.8±1.3	21.8±1.0	21.4±1.1	19.9±1.1
	right(n = 8)	20.1±0.8	21.0±0.9	22.2±1.0	21.2±1.7	20.3±0.9
	total(n = 16)	20.2±0.8	20.9±1.1	22.0±1.0	21.3±1.4	20.1±1.0
Am(°)	left(n = 9)	39.3±2.0	41.5±2.4	42.3±2.3	39.7±1.7	37.1±2.1
	right(n = 9)	38.8±1.8	41.9±2.7	42.4±2.8	39.8±2.2	37.6±1.9
	total(n = 18)	39.1±1.8	41.7±2.5	42.3±2.5	39.7±1.9	37.4±1.9
Ag(°)	(n = 9)	-7.7±7.4	-2.4±7.0	6.3±5.5	13.4±5.5	20.7±6.3
H1(mm)	left(n = 9)	1.0±2.8	2.3±1.1	2.4±0.5	2.7±1.5	3.2±1.1
	right(n = 9)	-0.1±3.4	1.7±0.8	3.1±1.4	2.8±1.3	2.5±1.0
	total(n = 18)	0.5±3.1	2.0±1.0	2.8±1.1	2.8±1.3	2.9±1.1
H2(mm)	left(n = 9)	12.6±2.9	12.4±1.8	12.2±1.3	12.7±1.7	12.9±2.3
	right(n = 9)	13.3±1.5	11.5±0.8	12.4±1.1	12.0±1.2	12.3±1.8
	total(n = 18)	12.9±2.2	12.0±1.4	12.3±1.2	12.3±1.5	12.6±2.0

(D) Vertical distance between entry point and the midline, (Am) Pedicle transverse angle, (Ag) Intra-operative pedicle sagittal angle, (H1) Vertical distance between the LPA and inferior margin of upper lateral mass, (H2) Vertical distance between the LPA and inferior margin of lateral mass itself.

All parameters varied according to the different spine levels (i.e., C3–C7).The Distance (D) was smallest at C7 (20.1±1.0mm) and largest at C5 (22.0±1.0mm).The Am was smallest at C7 (37.02±2.6°) and largest at C5 (42.34±2.52°).The Ag was approximately in the cranial orientation at C3–4 and in the caudal orientation at C5–7, with large dispersion.H1 was near 2–3mm at C4–7 and extremely variable at C3 (range -5.2–3.6mm). H2 was approximately 12–13mmat C3–7.However, there was no significant difference in these parameters between the bilateral pedicles (except for Ag) at each level using the paired t-test (*P* > 0.05).The date of L1 and L2 was omitted for total subjectivity depending on different observers.

Post-operative screw positions on the CT scans are displayed in Table 2. Overall, 78 (88.6%) of the 88 screws were considered as being in the correct position (grade 0), 4 (4.5%) screws were considered as achieving non-critical perforation (grade 1), and 6 (6.8%) screws were considered as achieving critical perforation (grade 2, 3). The 6 critical misplacements (grade 2, 3) were located at C4 in 3cases, at C6 in 1 case, and at C7 in 2 cases. Of the10 (11.4%) screws that were classified as causing perforation, 9 perforated the pedicle lateral wall and 1 perforated the inferior wall. Superior or medial screw perforations were not observed in any case.

**Table 2:** Post-operative screw position and perforation orientation.

	C3 (%)	C4 (%)	C5 (%)	C6 (%)	C7 (%)	Total (%)
Grade 0	15(93.8)	15(83.3)	17 (94.4)	16(88.9)	15(83.3)	78(88.6)
Grade 1	1(6.3)	0	1(5.6)	1(5.6)	1(5.6)	4(4.5)
Grade 2	0	2(11.1)	0	0	2(11.1)	4(4.5)
Grade 3	0	1(5.6)	0	1(5.6)	0	2(2.3)
Lateral wall perforation	1(6.3)	2(11.1)	1(5.6)	2(11.1)	3(16.7)	9(10.2)
Medial wall perforation	0	0	0	0	0	0
Superior wall perforation	0	0	0	0	0	0
Inferior wall perforation	0	1(5.6)	0	0	0	1(1.1)

## Discussion

During CPS insertion with fluoroscopy, the cervical pedicle wall perforation rate was reported to range from 6.7% to 29.8%[5, 20–23].Cong et al. conducted 10% (9/90) screws breached the pedicle cortex using the uniplanar locator[24]. In the current study, 4 out of 88 screws (4.5%) achieved non-critical perforation (grade 1), and 6 out of 88 screws (6.8%) achieved critical perforation (grade 2, 3).The perforation rates were roughly similar to previous studies using freehand techniques with the assistance of fluoroscopy or home-made instrument.

Screw misplacements were significantly more frequent at C4 (3/10 misplacements) and C7 (3/10), but less common at C3 (1/10), C5 (1/10), and C6 (1/10). The majority misplacements (9/10) occurred laterally and no medial breach was detected, which was consistent with previous reports showing that injuries mostly involved the vertebral artery [8, 21, 25].

In two of the grade 3 misplacement cases, the entry point was located correctly and the pilot hole was prepared correctly, however, the authors omitted the tapping step, and inserted the screw directly. Without the fine thread created by tapping, the entrance of the pilot hole worked against the initial insertion of the screw, which resulted in a serious breach into the transverse foramen, leaving the prepared channel empty (Fig 4D). Therefore, if every step (i.e., drilling, detecting, tapping, and screwing) was obligatory, the same mistake could be avoided in future by adopting a standard operation procedure.

**Fig 5:**
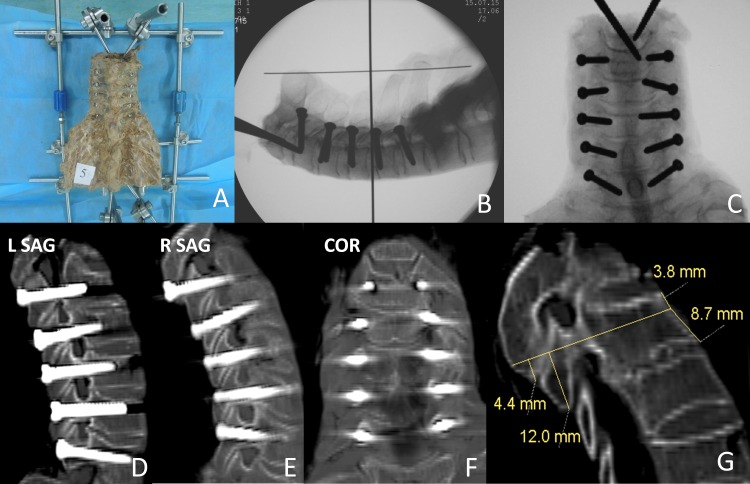
Imaging studies of an illustrative case (1). (A) A specimen was fixed by external fixator. (B and C) Intra-operative lateral view and post-anterio view. (D–F) Postoperative oblique sagittal and coronal image of CT shows good CPS placement (Grade 0). (G) As ideal entry point was obscured by proliferated inferior articular process of upper vertebra, probe must penetrate three layers bone cortex into the true cavity.

Deformed landmarks are often found intra-operatively in spinal degenerative disease or severe fracture dislocation, which makes morphological markers inaccurate when determining the ideal entry point[7–9, 11, 12, 26].For example, when the ideal entry point is obscured by proliferated inferior articular process of the upper vertebra, the probe must penetrate three layers bone cortex to get into the true cavity (Fig 5G). Thus, the traditional methods of measuring or observing anatomical landmarks of target vertebra is not feasible in these cases, as the true starting point on the upper vertebra is only locatable in the preoperative oblique sagittal plane. Instead, direct exposure techniques with enlarged entrance holes, a gutter, and laminoforaminotomy were advocated, which are accompanied by extra bone loss of lateral mass [1, 27, 28].In this study, with the help of caliper A, the entry point on the lateral mass was defined individually by measuring the D,H1and H2, which prevents extra bone loss. Moreover, caliper A helped us to determine the entry point objectively instead of subjectively.

To our knowledge, this is the first report to measure the sagittal angle of a pedicle during operation referring to the gravity line, which was usually ambiguously described as upward or downward[7, 11]. The superior or inferior vertebral endplate had previously been chosen as the reference line for preoperative measurements, but it is useless during operations due to variations in the spine level, physiological curvature, and operative position[7, 10–12, 15].The authors recommended the gravity line as a credible reference line to measure the real-time sagittal angle of the pedicle in the lateral fluoroscopic view with free hanging K-wires. The Ag read out from the goniometer (Fig 1B–1F) was not as accurate as the Am measured by software in the CT image(Fig 1A); however, it was easy to obtain for every segment, and could be read in real-time before the last insertion, thereby excluding most interfering factors. Using the mean value of multiple measurements may be more precise for the foremost placement.

Since the difference of 1–5°is difficult to discern intra-operatively, judgment of tridimensional drilling angles (Am and Ag) with the naked eye in traditional freehand techniques is prone to cause orientation mistakes. In our technique, Ag was realized by using the sliding bar of caliper B (Fig 3C and 3D) together with Am (Fig 3A and 3B), correct to 1°. The ideal triaxial screw channel was prepared along the connecting line between the intersection of the sliding bars and the entry point using caliper B (Fig 3E).

Considering that the medial pedicle cortex is thicker than the lateral cortex and that vertebra rotation tends to oppose pushed by the oblique puncture force, the drill was suggested to be inserted along the lateral direction of the sliding bar, and toward the medial cortex to lessen lateral perforation of the CPS (Fig 2B)[2, 6, 12, 25]. Moreover, as the entrance and orientation of the pedicle were unique at each cervical level, surgeons are advised to design and insert each CPS individually [8, 9, 12, 29].

**Fig 6:**
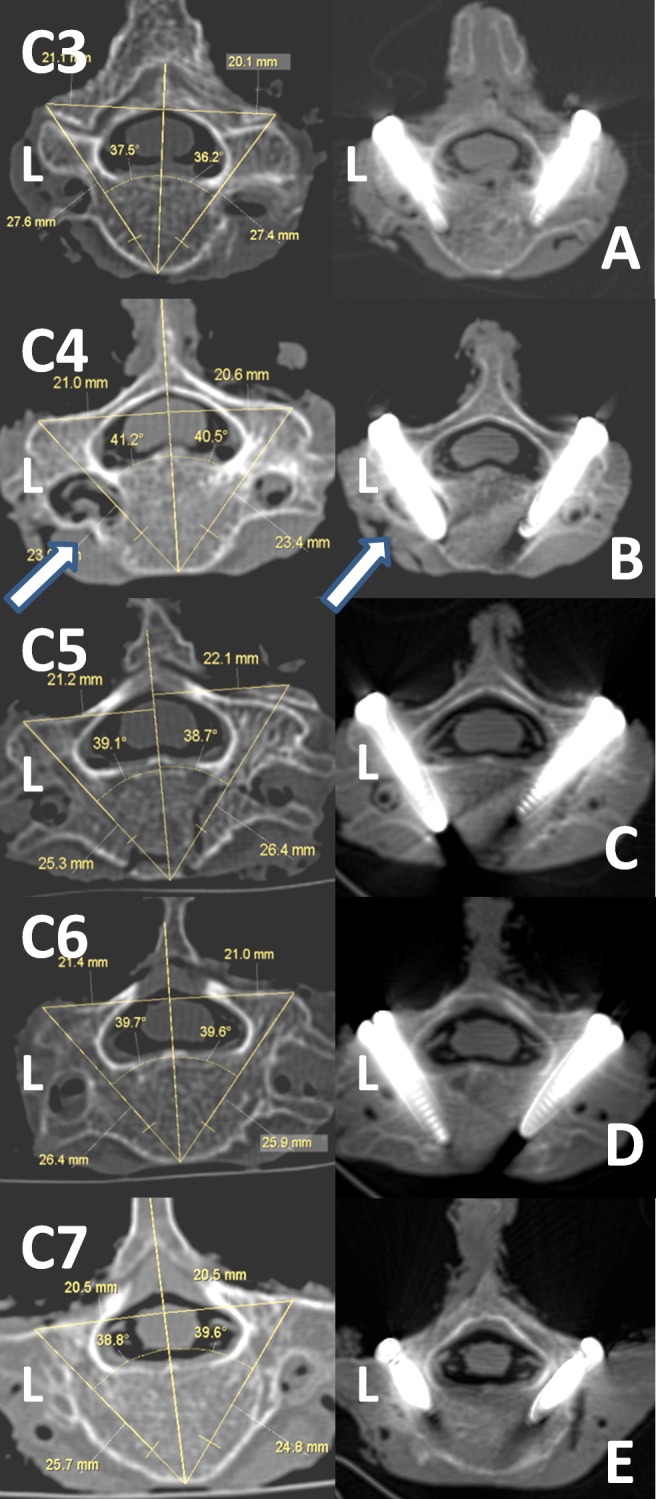
Imaging studies of an illustrative case (2). **(**A–E) Per-operative measure (left) and postoperative (right) axial image of CT. (B) Difference of diameter in left and right pedicles in C4 vertebra (white arrow). Although CPS was inserted in ideal orientation, injury to the vertebral artery was probable unavoidable.

Figs 5 and 6 have shown the serial images of typical screw placement procedure. It should be taken into account that parameters in this study were used for operative design and insertion, which are not absolutely equal to the theoretical pedicle trajectory in anatomical studies, especially in the C7 pedicle or relatively larger C3–6 pedicles[23]. Besides, a distinctly narrow pedicle observed preoperatively may preclude transpedicular fixation, and avoid probable perforation. Even if the CPS was placed in ideal orientation in these patients, injury to the dominant vertebral artery would be unavoidable because of the malformed pedicle (Fig 6B, white arrow).

Compared with other assistance tools or navigation systems, the novel calipers have additional advantages; they are cheap, easy to clean, reusable, sterilizable, and have a low technical demand. Therefore, caliper A and B could be useful assistant tools to master the transpedicular fixation technique for less experienced surgeons. Moreover, as Yoshimoto et al. described an obvious decrease in the perforation rate from 12% to 1.1% during three continuous periods of CPS insertion, novice surgeons should be supervised by experienced instructors to avoid risky complications, while understandings of morphologic features of pedicles and tactile feel during the drilling are still emphasized[20, 25]. If the equipment and technical conditions permitting, more accurate navigation systems (e.g., O-arm navigation, Iso-C 3D navigation, CT-based navigation, the patient-specific template system and the robotic system) would be better choice for complex CPS insertion certainly. Lastly, as perforation of CPS was not completely eliminated in this procedure, a combination of calipers with other free-hand or navigation techniques may lead to better surgical outcome.

### Limitations

It was impossible to differentiate whether the Ag was for either pedicle from a lateral fluoroscopic image. Therefore, using the C-arm to check the pedicle axis view for bilateral pedicles could be a critical supplement to ensure safe CPS placement [21, 23]. In addition, clinical trials on the safety and possibility of CPS insertion with caliper A and B should be performed.

## Conclusions

By means of the gravity line, the novel caliper A and B were helpful for locating the ideal entry point and guiding the individual stereoscopic insertion of cervical pedicle screws.

## Supporting information

S1 File Relevant parameters for preoperative design. (XLSX)

S2 File Postoperative screw position and perforation orientation. (XLSX)

S3 File CT scan for all samples. (RAR)
