# Correction: *In vitro* study of accuracy of subaxial cervical pedicle screw insertion using calipers based on the gravity line

**DOI:** 10.1371/journal.pone.0314788

**Published:** 2024-11-27

**Authors:** Xiang Yao, Shiqing Liu

An additional affiliation is missing for the first author. Xiang Yao is also affiliated with the Department of Orthopedics, The Affiliated People’s Hospital of Jiangsu University, Zhenjiang, Jiangsu Province, PR China.
